# Preoperative and Postoperative Physical and Mechanical Rehabilitation Interventions in Hallux Valgus: A Systematic Review

**DOI:** 10.1002/jfa2.70083

**Published:** 2025-09-11

**Authors:** Oya Gumuskaya, Benjamin Peterson, Hailey Donnelly, Banu Unver, Damien Lafferty, Peta Tehan

**Affiliations:** ^1^ School of Health Sciences Faculty of Medicine and Health University of Sydney Sydney Australia; ^2^ School of Nursing and Midwifery College of Health, Medicine and Wellbeing University of Newcastle Newcastle Australia; ^3^ School of Nursing and Midwifery Western Sydney University Sydney Australia; ^4^ Department of Podiatry School of Health, Medical and Applied Sciences Central Queensland University Rockhampton Australia; ^5^ School of Health Sciences College of Health Medicine and Wellbeing University of Newcastle Newcastle Australia; ^6^ Physical Therapy and Rehabilitation Faculty of Health Sciences Lokman Hekim University Ankara Turkey; ^7^ Department of Surgery School of Clinical Sciences Monash University Victoria Australia

**Keywords:** bunion, hallux valgus, orthotic, perioperative care, rehabilitation, surgery

## Abstract

**Background:**

Approximately one‐third of the adult population is affected by hallux valgus (HV). Surgical interventions are successful in reducing deformity; however, postoperative complications are common. There is growing evidence for prehabilitation and rehabilitation strategies in orthopaedic surgeries. However, the effectiveness of such strategies in HV surgery is currently unknown. This systematic review aimed to synthesise and determine the quality of evidence for the effectiveness of physical and mechanical prehabilitation and postoperative rehabilitation interventions for improving outcomes following HV surgery.

**Methods:**

Electronic databases: MEDLINE, Cochrane, CINAHL, Scopus, EMBASE and AMED were searched from inception until 19th May 2025, following the PRISMA guidelines. Randomised controlled trials were included to determine the effectiveness of preoperative and postoperative physical and mechanical therapies for improving outcomes in adults undergoing HV surgery. The evidence from individual studies was narratively synthesised, and data were not pooled because of the heterogeneity of interventions, methods and outcomes measures.

**Results:**

A total of 8166 titles and abstracts were screened, and 66 full‐text papers were reviewed. Five studies met the eligibility criteria and were included in this review. No randomised controlled trials examined the effectiveness of eligible preoperative physical or mechanical interventions. Postoperative early weight‐bearing, dynamic metatarsal splinting and transcutaneous ultrasound appeared to improve patient outcomes, whereas rigid‐soled footwear improved patient satisfaction.

**Conclusion:**

There is currently no evidence to support the effectiveness of preoperative physical and mechanical interventions for improving outcomes in HV surgery, and limited evidence supports postoperative interventions. Future trials should consider incorporating validated outcome measures.

## 1 Introduction

Hallux valgus (HV) is a joint disorder impacting up to 30% of adults, with greater prevalence in older females [1, 2, 3]. Typically characterised by lateral deviation of the hallux towards the lesser toes and an increased intermetatarsal angle, resulting in the first metatarsal head becoming more prominent and the first metatarsophalangeal joint (MTPJ) becoming increasingly mal‐aligned [1]. The MTPJ can progressively sublux and alter foot function as a result. Consequently, lesser toes can deform, causing disruptions to gait and plantar pressure distribution [4]. As HV progresses, individuals commonly experience pain, difficulty with footwear, corns and calluses and disturbed body image [1]. The severity of HV is also associated with reduced health‐related quality of life and function [5]. As a result, individuals with HV commonly seek surgical correction, one of the most commonly performed orthopaedic procedures [2].

Surgical correction has been demonstrated to be superior to conservative options for reducing HV angle [1, 5, 6]. Over 150 different surgical procedures have been described in the literature. More commonly used surgical methods include Akin, Chevron, scarf, Hohman osteotomy, metatarsophalangeal joint arthrodesis, Hueter or Keller‐Brandes arthroplasty [7]. However, the superiority of one surgical technique over another has yet to be demonstrated. Following HV corrective surgery, dissatisfaction is common, and complications can include recurrence of deformity, infection, non‐union or malunion, complex regional pain syndrome, deep vein thrombosis, fracture, osteonecrosis and hallux varus [8].

Prehabilitation and rehabilitation, used extensively in orthopaedic surgery, are designed to reduce complications, and improve outcomes [9, 10, 11]. It is proposed that these preoperative gains in strength and range of motion lead to improved postoperative outcomes [9, 10, 11]. Evidence demonstrates that prehabilitation decreases hospital length of stay in older adults undergoing major joint replacement and improves functional recovery [12, 13, 14, 15]. Similarly, rehabilitation has been demonstrated to improve clinical outcomes in other orthopaedic surgeries [10, 11, 16]. Whilst there is growing evidence for prehabilitation and rehabilitation in shoulder, knee and hip arthroplasty, the clinical utility of prehabilitation or rehabilitation in HV surgery has not been adequately investigated [17, 18].

This systematic review aimed to identify and determine the quality of evidence for the effectiveness of physical and mechanical prehabilitation and postoperative rehabilitation interventions for improving outcomes following HV surgery. This evidence will inform perioperative care practices in individuals undergoing HV surgery.

## 2 Methods

### 2.1 Search Strategy

This systematic review was conducted in accordance with the Preferred Reporting Items for Systematic Review and Meta‐Analysis (PRISMA) guideline. The review was prospectively registered on PROSPERO with registration number CRD42022329933. One deviation from the protocol was made, reported under the heading exclusion criteria. An electronic search of MEDLINE, CINAHL, Cochrane, Scopus, Embase and AMED databases was performed from inception to 19th May 2025. The search strategy applied to MEDLINE is presented in Appendix 1 and was adapted for each database. Retrieved records were exported into Covidence [19]. Two authors independently screened titles and abstracts (OG, HD or PT), with a third author arbitrating disagreements (BP). Two authors (PT and OG) independently screened full‐text studies with arbitration by a third author (BP). Reference lists of included articles were manually screened for any additional records not identified during electronic database searches.

### 2.2 Inclusion Criteria

All randomised controlled trials (RCTs) of physical and mechanical therapies for improving postoperative outcomes among adults undergoing HV corrective surgery were eligible for inclusion. Trials were included regardless of participant blinding.

### 2.3 Exclusion Criteria

Studies including paediatric populations and juvenile HV were excluded. Studies which used combined interventions were excluded if the effect of the physical and mechanical intervention could not be determined.

At the time of PROSPERO registration, the protocol outlined that RCTs, quasi‐RCTs and cohort studies would be included. After registering this systematic review, but before commencing study screening, the decision was made to limit the eligibility criteria to RCTs (therefore excluding quasi‐RCTs and cohort studies), to better reflect the scope and purpose of the current review.

### 2.4 Interventions

Eligible interventions were non‐invasive physical or mechanical therapies for improving post‐surgical outcomes among adults undergoing HV corrective surgery. Physical therapies could include but were not limited to stretching, strengthening, or mobilisation exercises. Mechanical interventions could include but were not limited to splinting, bracing, footwear or foot orthoses. Invasive therapies, including but not limited to pharmacological interventions, surgical procedures or dry needling were ineligible for inclusion. Interventions could be implemented either preoperatively or postoperatively.

### 2.5 Outcome Measures

The primary outcome measures were pain severity or duration, and health‐related quality of life. The secondary outcomes included: functional ability, the length of postoperative hospitalisation, return to weight‐bearing activities, return to recreational and sporting activities, rates of any postoperative complications (e.g., pain, reoccurrence of deformity), days to wound healing and requirements for pharmacological or other forms of pain management, as well as adverse events or satisfaction associated with the application of the intervention.

### 2.6 Data Extraction and Analysis

Data were extracted from eligible studies using a standard data extraction tool, which was developed and pilot tested for this review and included the following: study design, eligibility criteria, recruitment procedures, setting, interventions, outcome measures, follow‐up duration, power calculation, baseline participant characteristics, outcome data and funding source. Data were extracted by one reviewer (BU) and checked by a second reviewer (BP or OG). Authors from the included studies were to be contacted for clarification in cases of missing or ambiguous data, but this was not required. The results of included studies were reported in this review by means of narrative synthesis. Outcomes of interventions reported within the individual included studies were descriptively sub‐grouped according to the type of intervention in the narrative synthesis.

### 2.7 Risk of Bias

To assess the potential sources of bias among included studies, the Cochrane risk of bias version 2 (ROB‐2) tool was utilised by two reviewers independently (HD and OG) with arbitration by a third reviewer (BP) [20].

## 3 Results

**FIGURE 1:**
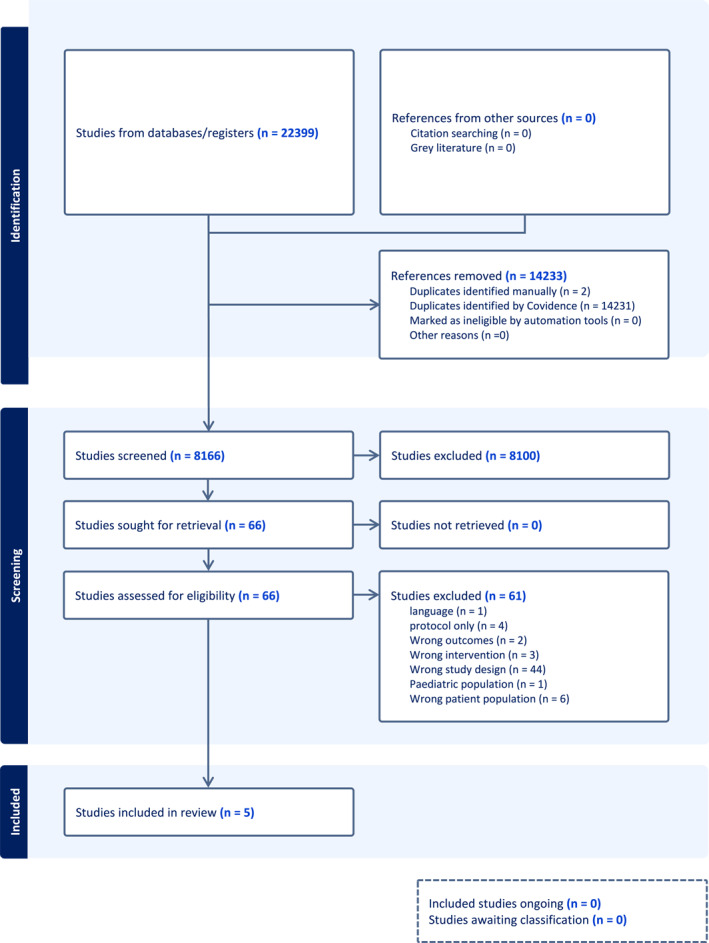
PRISMA flow diagram.

A total of 8166 titles and abstracts were screened for eligibility after removing duplicates, with 66 full‐text studies reviewed. Five studies met the eligibility criteria and were included (Figure 1).

### 3.1 Characteristics of Included Studies

A total of 279 participants were included in the five trials, ranging from *n* = 39 to *n* = 90 (Table 1). The mean participant age range across the studies was 40–64 years, with two of the five studies not reporting age [23, 24]. Participants were predominantly women in most studies, with between 52% and 94% of participants identified as female, with two studies not reporting sex [21, 22]. Two of the studies were from the United States of America, one from Australia, one from Austria and one from China. Three of the included studies were conducted in an outpatient setting, one was within the participant's home and one was in a hospital setting. Two studies included paired data with both limbs of participants included, whereas the remaining studies did not specify.

**TABLE 1:** Study characteristics.

Study	Study design	Country	Setting	Control (n)	Intervention (n)	Total (n)	Number of feet	Female (%)	Mean age	
									Control	Intervention
Connor et al. (1995) [21]	RCT	United States	Home/after discharge	18	21	39	Not specified	Not specified	41.1 ± 13.4	40.7 ± 13.3
Dearden et al. (2019) [22]	RCT	Australia	Outpatient hospital setting	47	43	90	Not specified	Not specified	64 (27–84)	61.1 (27–84)
John et al. (2011) [23]	RCT	United States	Outpatient hospital setting	23	25	48	Not specified	52%	Not specified	Not specified
Ling et al. (2020) [24]	RCT	China	Outpatient	29	21	50	50	90%	Not specified	Not specified
Zacherl et al. (2009) [25]	RCT	Austria	Hospital	Not specified	Not specified	44	52	85%	54 (28–77)	51 (20–77)

Abbreviations: n, number; RCT, Randomised controlled trial.

### 3.2 Surgical Interventions

The methods for HV surgery included the Austin procedure [21], Scarf and Akin osteotomy or first MTPJ arthrodesis [22], endoscopic‐assisted digital soft‐tissue procedures [24] and Chevron osteotomy [25]. The method of foot surgery was not explicitly reported in one trial [23]. Dearden et al. (2019) and Connor et al. (1995) reported a single surgeon performed the procedures, Zacherl et al. reported that the surgical method was standardised for all participants, and Ling et al. reported that different surgeons performed the procedures in a single centre (Table 2).

**TABLE 2:** Study selection criteria, surgery type and interventions.

Study (year)	Selection criteria		Surgery type	Control	Intervention
	Inclusion	Exclusion			
Connor et al. (1995) [21]	Adults undergoing an Austin procedure to correct HV	None	Austin procedure	Physical therapy (self‐assisted PROM and active ROM + hydrotherapy)	Continuous passive motion + physical therapy (self‐assisted PROM, active ROM + hydrotherapy)
Dearden et al. 2019) [22]	Patients undergoing additional forefoot procedures at the same time as scarf and Akin osteotomies First MTPJ arthrodesis (such as interphalangeal joint arthrodesis of lesser toes, soft tissue corrective procedures and excision of Morton's neuroma)	Bilateral surgery, revision procedures and additional arthrodesis Osteotomy procedures being performed proximal to the forefoot, Cognitive impairment to follow postoperative instructions or consent	Scarf/Akin osteotomies or first MTPJ arthrodesis	Reverse camber shoe	Rigid flat‐sole shoe
John et al. (2011) [23]	Diagnosed with hallux limitus following surgery, Reduced flexibility in active range of motion of extension in the great toe. Pain that is worsened by walking and/or squatting. Impaired gait pattern.	Metatarsal stress fracture. Interdigital neuroma. Sesamoid pathology. Gout. Metatarsalgia.	Surgical procedures for foot pathologies.	Standard care	Metatarsophalangeal dynasplint (extension) system with standard care (analgesics and NSAIDs, orthotics, and home stretching exercises)
Ling et al. (2020) [24]	Undergoing the endoscopic‐assisted distal soft tissue procedure for HV correction. Mentally and legally capable of consent to participate in this study.	Disabilities (both physical and mental) which may impair the adherence of the rehabilitation Those who have concomitantly undergone additional procedures on the same foot.	Endoscopic‐assisted distal soft tissue procedure for HV correction.	Existing protocol of non‐weight‐bearing walking for 6‐week before the resumption of partial‐weight‐bearing walking for 6‐week then full‐weight‐bearing walking at 12‐week post‐surgery.	Early weight‐bearing; 2‐week of non‐weight‐bearing following surgery followed by 10‐week of partial‐weight‐bearing and resumption of full‐weight‐bearing walking at 12‐week after surgery.
Zacherl et al. (2009) [25]	Patients admitted for surgical treatment (chevron osteotomy) of symptomatic mild to moderate HV deformity	Other types of hallux surgery Previous surgery or any bony lesion on the affected foot Planned additional surgery on the same foot A known history of poor bone formation Treatment with antiresorptive drugs Body mass index over 36 Inclusion in another study	Chevron osteotomy	Placebo ultrasound treatment along with use of wedge‐based Postoperative shoe and redressing Bandage	Transcutaneous low intensity pulsed ultrasound treatment along with use of wedge‐based Postoperative shoe and redressing Bandage

Abbreviations: HV, Hallux valgus; MTPJ, Metatarsophalangeal joint; NSAID, Non‐steroid anti‐inflammatory drugs; PROM, Passive ROM; RCT, Randomised controlled trial; ROM, Range of motion.

### 3.3 Description of Physical Rehabilitation Interventions and Outcomes

A range of intervention and outcomes were evaluated among included studies.

### 3.4 Interventions for Improving Joint Range of Motion

Two studies evaluated the effectiveness of interventions for improving joint range of motion (ROM) postoperatively [23]. Connor et al. (1995) compared the effect of continuous passive motion (in addition to the self‐assisted passive/active range of motion of the first MTPJ and hydrotherapy) against no continuous passive motion (with self‐assisted passive and active range of motion of the first MTPJ and hydrotherapy) on the postoperative range of motion of the first MTPJ at day 7 and day 90. John et al. (2011) evaluated the effectiveness of a metatarsophalangeal dynasplint (extension) system plus standard care (analgesics and NSAIDs, orthotics and home stretching exercises) versus standard care alone on postoperative active first MTPJ active dorsiflexion ROM over an eight‐week intervention period (Tables 2 and 3).

**TABLE 3:** Study outcomes and results.

Study (year)	Control	Intervention	Primary Outcome Measurement	Follow‐up duration		Baseline	Follow‐up 1	Follow‐up 2
Connor et al. (1995)^a^ [21]	Physical therapy (self‐assisted PROM and active ROM + hydrotherapy)	Continuous passive motion + physical therapy (self‐assisted PROM, active ROM + hydrotherapy)	Mean MTPJ extension ROM	Day 7 and 90.	Control	61.7	(70% of intraoperative ROM) 43.3 ± 3.8	85% of intraoperative ROM
					Intervention	64.5 ± 6.10	(90% of intraoperative ROM) 58.3 ± 6.4	96% of intraoperative ROM
Dearden et al. (2019) [22]	Reverse camber shoe	Rigid flat‐sole shoe	Postoperative VAS pain score (0–100 point score; higher score indicates more severe symptoms)	Six weeks	Control	—	30.9 (range, 0–83)	—
					Intervention	—	26.4 (range, 0–80)	—
John et al. (2011) [23]	Standard care	Metatarsophalangeal dynasplint (extension) system with standard care (analgesics and NSAIDs, orthotics and home stretching exercises)	Active dorsiflexion ROM at first metatarsal joint	Eight weeks	Control	—	10°	—
					Intervention	—	32°	—
Ling et al. (2020) [24]	Existing protocol of non‐weight‐bearing walking for 6‐week before the resumption of partial‐weight‐bearing walking for 6‐week then full‐weight‐bearing walking at 12‐week post‐surgery.	Early weight‐bearing; 2‐week of non‐weight‐bearing following surgery followed by 10‐week of partial‐weight‐bearing and resumption of full‐weight‐bearing walking at 12‐week after surgery.	Foot and ankle outcome score (0–100 point score; lower score indicates more severe symptoms)	12 and 26 weeks	Control			
					Symptoms	58.3 ± 26.1	62.1 ± 16.1	81.9 ± 11.8
					Pain	44.0 ± 23.8	66.4 ± 19.3	82.2 ± 15.6
					Activity of daily living	59.7 ± 29.4	61.4 ± 17.3	84.3 ± 10.6
					Sport	52.1 ± 29.7	n/a	76.7 ± 15.9
					Quality of life	41.2 ± 30.2	49.1 ± 20.4	82.4 ± 11.2
					Intervention			
					Intervention Symptoms	47.5 ± 23.9	76.9 ± 13.6	80.5 ± 15.6
					Pain	47.5 ± 28.8	78.6 ± 11.4	85.5 ± 12.9
					Activity of daily living	53.0 ± 25.6	81.4 ± 7.6	86.0 ± 8.2
					Sport	39.8 ± 26.7	n/a	77.6 ± 13.7
					Quality of life	31.1 ± 23.6	72.6 ± 20.7	81.4 ± 14.9
Zacherl et al. (2009) [25]	Placebo ultrasound treatment along with use of wedge‐based postoperative shoe and redressing Bandage	Transcutaneous low intensity pulsed ultrasound treatment along with use of wedge‐based postoperative shoe and redressing Bandage	The American orthopaedic foot and ankle Society's hallux‐metatarsophalangeal‐interphalangeal scale (0–100 point score; lower score indicates greater symptoms or impairments) Total flexion and extension ROM at the first metatarsal joint	Six weeks (intervention only) and one year	Control n	Baseline 26		One year follow up 28
					HV angle (degrees)	31 (8.7)		16 (9.2)
					Intermetatarsal angle (degrees)	13 (2.5)		8 (2.8)
					Sesamoid position index (0–3)	2.3 (0.8)		−2 (2.4)
					Distal metatarsal articular angle (degrees)	8 (6.2)		8 (5.8)
					Arthrosis of the first MTPJ, according to kellgren (0–4)	0.9 (0.8)		1.8 (0.8)
					Intervention n	Baseline 26		One year follow up 26
					HV angle (degrees)	31 (8.7)		19 (8.4)
					Intermetatarsal angle (degrees)	12 (2.7)		8 (2.2)
					Sesamoid position index (0–3)	2.0 (0.9)		−3 (2.1)
					Difference in length of first and second metatarsal (mm)	0 (1.9)		10 (4.1)
					Distal metatarsal articular angle (degrees)	10 (4.3)		1.1 (0.9)
					Arthrosis of the first MTPJ according to kellgren (0–4)	0.5 (0.7)		1.8 (0.8)

Abbreviations: HV, Hallux valgus; mm, millimetres; MTPJ, Metatarsal phalangeal joint; n, number; NSAID, Non‐steroid anti‐inflammatory drugs; PROM, Passive ROM; ROM, Range of motion.

^a^ The study had an additional four follow‐up outcome measurement time points (days 14, 21, 28, and 60) that have not been included in this table.

### 3.5 Interventions to Reduce Pain

One study evaluated the effect of interventions for improving postoperative pain. Dearden et al. (2019) compared the effectiveness of reverse‐camber footwear versus rigid flat‐soled footwear on postoperative pain rating, measured using a 100 mm visual analogue scale (VAS) over a six‐week intervention period (Table 2).

### 3.6 Interventions for Physical Functioning

The study by Ling et al. (2020) investigated the effect of an early weight‐bearing intervention compared with non‐weight‐bearing for 6 weeks before the resumption of partial‐weight‐bearing walking at postoperative 6‐week, then full‐weight‐bearing walking at 12‐week. Outcomes were the foot and ankle outcome score at postoperative 12‐week and 26‐week. The early weight‐bearing intervention used by Ling et al. (2020) included two weeks of non‐weight‐bearing followed by ten weeks of partial‐weight‐bearing and resumption of full‐weight‐bearing walking at 12‐week (Table 2).

### 3.7 Interventions for Correction of Deformity

Zacherl et al. (2009) explored the utilisation of postoperative daily transcutaneous low‐intensity ultrasound treatment in combination with a wedge‐based postoperative shoe and a redressing bandage compared to a placebo ultrasound treatment in combination with a wedge‐based postoperative shoe and a redressing bandage, for 6 weeks following Chevron osteotomy. Plain dorsoplantar radiographs, hallux‐metatarsophalangeal‐interphalangeal scale and a questionnaire on patient satisfaction measures were collected at 6 weeks and at 1‐year post‐surgery to assess intervention effectiveness (Table 2).

### 3.8 Quality Appraisal

**FIGURE 2:**
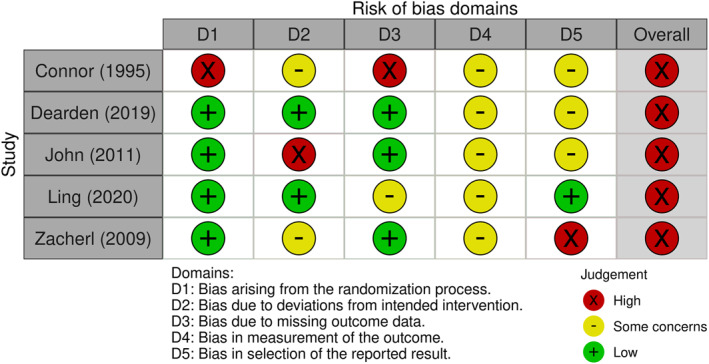
Risk of bias.

All five included studies returned a high risk of bias following quality appraisal using the revised Cochrane risk‐of‐bias tool for randomised trials (RoB 2) 2019 version [20]. Common sources of bias related to deviations from the intended intervention, measurement of outcomes and selective outcome reporting. The results of the quality appraisal of the five included studies are reported in Figure 2 [26].

The allocation sequence concealing prior participant enrolment to interventions or the evidence regarding all outcomes presented was not demonstrated in the Connor et al. study [21]. The clinicians delivering the metatarsophalangeal dynasplint or continuous passive motion + physical therapy (self‐assisted prom and active rom + hydrotherapy) were not able to be blinded in the John et al. study, and the study was funded by the manufacturer of the device they were evaluating, which is a potential source of bias [23]. There was no information to determine if the results were analysed in accordance with a pre‐specified plan, and there were multiple outcome measurements used for the primary outcome indicating that results may have been selected based on results from multiple eligible outcomes or analyses in the Zacherl et al. study [25]. Both Dearden et al. and Ling et al. had ‘some concerns’ across multiple domains relating to outcomes which substantially lowered confidence in the results.

### 3.9 Effect of Interventions

Data relating to the effects of interventions of included studies are presented in Table 2. Data were presented as it is published in original studies. A meta‐analysis was planned; however, it was precluded by heterogeneity in the interventions, outcomes and statistical methods of included studies.

### 3.10 Continuous Passive Motion

Connor et al. (1995) found that the continuous passive motion and physiotherapy group had a significantly larger mean ROM (degrees) than the physiotherapy‐only group (58.3° ± 6.4 vs. 43.3° ± 3.8 *p* < 0.05), and a more extensive range of motion was gained more quickly over 90 days in seven follow‐ups postoperatively (8.71° vs. 11.86° for extension; *p* < 0.01; *n* = 39). The mean time to return to normal footwear was also significantly earlier in the continuous passive motion group compared to the physical therapy‐only group (*p* < 0.01) (Table 3).

### 3.11 Reverse Camber Shoe Versus Rigid Flat‐Soled Shoe

Dearden et al. (2019) identified no significant difference in postoperative pain among participants ambulating in a flat, rigid‐soled shoe (VAS 26.4; range 0–80) compared with participants ambulating reverse camber shoe (VAS 30.9; range 0–83) (*p* = 0.47). Dearden et al. (2019) did not identify any significant difference in overall participant satisfaction with the allocated footwear intervention (*p* = 0.089), satisfaction related to comfort (*p* = 0.32) or satisfaction related to pain relief when walking (*p* = 0.34). However, participants allocated to the rigid flat‐soled shoe were significantly more satisfied with their mobility (86% vs. 61%, *p* = 0.012), stability and risk of falls (90.7% vs. 69.6%, *p* = 0.027) compared to the reverse‐camber group. There was no postoperative stress fracture or non‐union recorded on postoperative 12‐week follow‐up x‐rays, and no differences in HV recurrence requiring revision surgery in either group at one‐year follow‐up. There was no difference in time‐to‐union; however, one participant in the rigid flat‐shoe had asymptomatic non‐union at postoperative nine months, and one case of superficial wound infection in each group required oral antibiotics (Table 3).

### 3.12 Metatarsophalangeal Dynasplint Versus Standard Care

John et al. (2011) studied the use of a dynamic splint (dynasplint) in conjunction with standard care versus standard care only, which included anti‐inflammatory drugs, orthotics and home exercise. There was a significantly greater improvement in active ROM for intervention versus control participants; 44° versus. 34° respectively (*p* = 0.001) in 48 individuals. A significant difference was also seen in the intervention group treated immediately (less than 2 months) following surgery versus participants treated more than 2 months following surgery (*p* = 0.0221) in twenty‐five individuals.

Two control group participants withdrew from the study by John et al. (2011) because of excessive pain. No adverse events were reported by the participants in the intervention group (Table 3).

### 3.13 Early Weight‐Bearing Versus Non‐Weight‐Bearing

Ling et al. (2020) studied early weight‐bearing compared to delayed weight‐bearing postoperatively. Comparison of the control and early weight‐bearing group showed no difference at the final time point (26‐week postoperative) in foot and ankle outcomes scores (FAOS), including symptoms, pain, activities of daily living, sport and quality of life. However, there were significant differences in foot function at the 12‐week postoperative in all subscales (*p* = < 0.01), excluding sporting activities, which was not recorded since most patients have not yet resumed sports. The accelerated 2‐week weight‐bearing group had significantly better symptoms (such as swelling, redness, or instability), less pain, better general function and improved quality of life at the 12‐week interval (*p* = < 0.01). Further, the intervention of early weight‐bearing walking at postoperative 2‐week did not increase implant failure (such as premature breakage/loosening of the 1,2 inter‐metatarsal positional screw) or recurrence rates (Table 3).

### 3.14 Transcutaneous Low‐Intensity Pulsed Ultrasound Versus Placebo Ultrasound

Zacherl et al. (2022) studied the effect of postoperative transcutaneous low‐intensity pulsed ultrasound versus placebo ultrasound following Chevron osteotomy and found no significant differences between the two groups for pain, range of motion or radiographic parameters (hallux–metatarsophalangeal–interphalangeal scale) (Table 3).

## 4 Discussion

This is the first systematic review to synthesise evidence from RCTs examining the effectiveness of preoperative and postoperative physical and mechanical interventions for improving outcomes in adults undergoing elective HV corrective surgery. Our findings highlight the lack of randomised trials in this area, with only five studies meeting the inclusion criteria, all focused on rehabilitation methods. Four of the five studies demonstrated a high risk of bias in relation to deviations from intended interventions, missing outcome data, measurement of the outcomes and selection of the reported results, demonstrating there is a need for more high‐quality trials in this area. Furthermore, this review did not elicit any literature on the effectiveness of prehabilitation for HV surgery. Whilst prehabilitation is a relatively new concept, there is a much larger evidence base in larger joint surgery [10, 11, 27]. The lack of research in this space highlights the current gap and justifies future trials in this area, with an aim to improve patient outcomes and reduce complications.

The heterogenous interventions in the trials in this review aimed to improve physical functioning, pain, and ROM or reducing joint deformity, with rehabilitation strategies ultrasound, splinting, physiotherapy, footwear and early weight‐bearing. Zacherl et al. (2022) demonstrated that low‐intensity ultrasound was ineffective at improving joint ROM, pain or radiographic parameters at 12th and 52nd week post Chevron osteotomy. The use of pulsed ultrasound has previously been proposed to accelerate fracture healing by increasing bone formation; however, it has not been demonstrated to accelerate functional recovery [28]. Whilst the underlying hypothesis of ultrasound to improve healing is accepted, inadequate evidence is currently available to support its use in rehabilitation post HV surgery [29].

Regarding the timing of postoperative weight‐bearing; one study [24] demonstrated postoperative complications (recurrence and screw displacement) did not increase significantly with early weight‐bearing. This provides some evidence dispelling concerns of starting early rehabilitation; however, should be interpreted with caution as it is a single study with a high risk of bias and outcomes beyond 26 weeks have not been investigated [24]. It is acknowledged that there is a large amount of variation in postoperative weight‐bearing recommendations following foot and ankle surgery [30], with limited available evidence to guide postoperative protocols. In other lower limb orthopaedic surgeries, there is more robust evidence to support early versus late weight‐bearing, which impacts on rehabilitation. For instance, a non‐randomised comparative study completed in 2019, demonstrated that early weight‐bearing after surgical fixation of distal femur fractures was associated with no greater risk of implant failure or fracture displacement, with delayed weight‐bearing associated with delayed fracture healing, and increased risk of fixation failure [31]. The findings of the trial by Ling (2020) suggest early weight‐bearing may assist with faster return to function following HV corrective surgery; however, more robust evidence is required including long term outcomes.

Stretching, strengthening and mobilisation are mainstay components of other orthopaedic postoperative rehabilitation programs [32]. However, there was a notable lack of studies completed on the use of stretching or strengthening as an intervention for improving outcomes following HV surgery. The current study found only two RCTs assessing the effectiveness of these interventions, with limited comparison possible because of heterogenous (active vs. passive) methods. One utilised passive ROM, and the other utilised a dynamic splint. The use of continuous passive ROM exercises saw faster return to ROM and normal footwear, along with a significantly larger mean ROM, compared to physiotherapy alone. Research completed in conservative (non‐surgical) management of HV has demonstrated similar positive outcomes. One study by Kim et al. (2015) utilised an orthosis with toe‐spread‐out exercise, which resulted in a reduction in HV angle at rest [33]. Another study in lower limb orthopaedic surgery investigated static progressive stretching post‐knee arthroplasty demonstrated that also resulted in significant increases in joint ROM and patient satisfaction [34]. Post HV surgery active joint ROM was also improved with the use of a dynamic splint. A previous study which examined the use of a dynamic splints post radial fractures also demonstrated a similar outcome, with an increase in active ROM at 3.9 weeks [35].

At commencement of postoperative weight‐bearing or semi‐weight‐bearing ambulation, specific footwear is frequently prescribed. There is evidence from one included study which points toward improved patient satisfaction related to general mobility, stability and risk of falls among participants using flat rigid‐soled shoes compared to reverse camber shoes [22]. However, no differences were demonstrated in postoperative pain or complications between footwear. It is noteworthy that the effectiveness of commercially available footwear for improving postoperative outcomes has not been explored. Specific footwear features, such as a rocker‐sole or a stiff‐soled shoe are widely used in the management of painful first metatarsophalangeal joint pathologies, such as structural hallux limitus or hallux rigidus [36]. Recently, Munteanu et al. (2021) demonstrated the effectiveness of carbon fibre shoe‐stiffening inserts for improving foot pain among individuals with first metatarsophalangeal osteoarthritis, and such commercially available and cost‐effective interventions for better outcomes following HV surgery may be warranted [37]. Menz et al. (2024) demonstrated that footwear and orthotic interventions significantly reduced peak pressure on the medial aspect of the first MTPJ, although no significant effects were observed at the interphalangeal joint. However, it remains uncertain whether these alterations in plantar pressure translate into meaningful improvements in symptoms associated with HV [38]. The use of commercially available footwear may also present challenges; a previous pilot trial reported low adherence rates, likely attributable to the physical appearance of the shoes [39]. Furthermore, women at increased risk of developing HV have been reported to compromise in their footwear choices, negotiating between functional comfort and the expression of personal identity [40]. The availability of suitable commercial footwear for postoperative HV rehabilitation remains unclear. As such, the feasibility and acceptability of commercial footwear as a rehabilitation strategy for individuals with HV may be limited at present.

### 4.1 Limitations

The limitations of the current review should be acknowledged. Whilst we conducted an exhaustive search of the literature, and utilised two individuals to screen all papers to reduce the likelihood of human error, it is possible that studies may have been missed. We did not contact researchers in the field for any unpublished studies. The scope of this review may have been limited by restricting its eligibility criteria to studies published in the English language. Heterogeneity in outcome measures and statistical supporting methods precluded pooled analyses of findings across studies, therefore the pooled effect of individual interventions could not be determined. To facilitate meta‐analysis in future systematic reviews, the authors recommend that authors of future RCTs of preoperative interventions for HV surgery report mean change from baseline data for all groups and outcome measures, with suitable measures of dispersion (e.g. standard deviation).

### 4.2 Clinical Recommendations

Prehabilitation has not been researched, therefore its current use in HV surgery is not known.

When considering rehabilitation post HV surgery, some interventions show promise, however included studies were of low quality, and included small sample sizes, so this should be considered when interpreting findings. To improve joint ROM, both continuous passive ROM, and the use of a dynamic splint post HV could be considered. To improve pain at 12 weeks post HV surgery, clinicians may cautiously consider early weight‐bearing. To improve patient satisfaction in relation to general stability, mobility and falls risk, consider the use of a stiff soled shoe.

## 5 Conclusion

Prehabilitation and rehabilitation are commonly employed to improve patient outcomes following orthopaedic surgery. This review aimed to determine the current RCT evidence for the effectiveness of prehabilitation and rehabilitation in HV surgery. No RCTs have evaluated the effect of prehabilitation on outcomes in HV surgery. Limited low quality evidence from two individual studies suggests the effectiveness of continuous passive ROM, and dynamic splinting improve joint range of motion following HV surgery. Additionally, the use of a flat rigid shoe following HV surgery was effective in improving patient satisfaction related to general mobility, stability and risk of falls. To establish the effectiveness of preoperative and postoperative interventions to improve HV surgery outcomes; future trials must demonstrate greater methodological rigour and standardised outcome reporting. Research is also needed to determine the optimal intensity, frequency and long‐term effects of rehabilitation protocols.

## Author Contributions

**Oya Gumuskaya:** conceptualization, formal analysis, investigation, methodology, project administration, supervision, validation, visualization, writing – original draft preparation, writing – review and editing. **Benjamin Peterson:** formal analysis, investigation, methodology, validation, writing – original draft, writing – review and editing. **Hailey Donnelly:** investigation, methodology, validation, writing – original draft, writing – review and editing. **Banu Unver:** formal analysis, investigation, methodology, validation, writing – review and editing. **Damien Lafferty:** writing – review and editing. **Peta Tehan:** formal analysis, investigation, methodology, project administration, supervision, validation, visualization, writing – original draft, writing – review and editing.

## Ethics Statement

The authors have nothing to report.

## Conflicts of Interest

The authors declare no conflicts of interest.

## Supporting information


Supporting Information S1

## Data Availability

Data were summarised in the evidence table.
