# Supplementary material for: Preoperative and Postoperative Physical and Mechanical Rehabilitation Interventions in Hallux Valgus: A Systematic Review
Source: J Foot Ankle Res. 2025 Sep 11;18(3):e70083. doi: 10.1002/jfa2.70083 (PMC12425807; doi:10.1002/jfa2.70083)
Supplement: Supplementary file 1 — Supporting Information S1 [file JFA2-18-e70083-s001.docx]

**Appendix 1. OVID Medline search strategy**

| 1 | Hallux valgus |
| --- | --- |
| 2 | Hallux abducto* |
| 3 | Bunion* |
| 4 | HV |
| 5 | HAV |
| 6 | 1 OR 2 OR 3 OR 4 OR 5 |
| 7 | Surg* |
| 8 | Operat* |
| 9 | Correct* |
| 10 | Interven* |
| 11 | Osteotom* |
| 12 | Scarf* |
| 13 | Austin* |
| 14 | Chevron* |
| 15 | 7 OR 8 OR 9 OR 10 OR 11 OR 12 OR 13 OR 14 |
| 16 | Rehab* |
| 17 | Prehab* |
| 18 | Exerci* |
| 19 | Post-op* |
| 20 | Post op* |
| 21 | Pre-op* |
| 22 | Preop |
| 23 | Therap* |
| 24 | Physio* |
| 25 | Physical |
| 26 | Mobil* |
| 27 | Orthos* |
| 28 | Orthot* |
| 29 | Boot* |
| 30 | Cast* |
| 31 | Shoe* |
| 32 | Footwear |
| 33 | Walker* |
| 34 | 16 OR 17 OR 18 OR 19 OR 20 OR 21 OR 22 OR 23 OR 24 OR 25 OR 26 OR 27 OR 28 OR 29 OR 30 OR 31 OR 32 OR 33 |
| 35 | 6 AND 15 AND 34 |
